# Outcomes and Associated Factors of Induction of Labor in East Gojjam Zone, Northwest Ethiopia: A Multicenter Cross-Sectional Study

**DOI:** 10.1155/2023/6910063

**Published:** 2023-06-14

**Authors:** Moges Agazhe Assemie, Getachew Tilaye Mihiret, Chernet Mekonnen, Pammla Petrucka, Temesgen Getaneh, Wassachew Ashebir

**Affiliations:** ^1^Department of Public Health, College of Health Sciences, Debre Markos University, Debre Markos, Ethiopia; ^2^Department of Midwifery, College of Health Sciences, Debre Markos University, Debre Markos, Ethiopia; ^3^Department of Midwifery, Debre Markos Comprehensive Specialized Hospital, Debre Markos, Ethiopia; ^4^College of Nursing, University of Saskatchewan, Saskatoon, Canada

## Abstract

**Background:**

Induction of labor is the initiation of uterine contractions by artificial methods once the fetus has reached viability and prior to spontaneous onset of labor with the aim of achieving vaginal delivery. Although induction of labor is a critical life-saving intervention that potentially reduces adverse pregnancy outcomes, sometimes it has undesirable consequences for the health of the mother and/or the fetus. Hence, this study aimed to evaluate the outcomes and associated factors of labor induction.

**Methods:**

An institution-based cross-sectional study was conducted from February 25 to May 25, 2020, among women undergoing induction at East Gojjam zone public hospitals in northwest Ethiopia. A structured interviewer-administered questionnaire was used to collect data from a sample of 411 mothers who were selected using a systematic random sampling technique. Stata/se™ Version 14 statistical software was used to analyze the data. Multivariable binary logistic regression was used to determine the potential factors affecting successful labor induction. Adjusted odds ratios with their 95% CI intervals were used to declare the strength of the association, and a variable with *p* value <0.05 was considered to have statistical significance.

**Results:**

The prevalence of successful induction of labor was 70.3% (65.6, 74.7). The favorable Bishop score ((CI 3.90, 1.63–9.29); *p* value = 0.002), the intermediate Bishop score ((CI 3.53, 2.15–5.82); *p* value = 0.001), labor induction using oxytocin with cervical ripening ((CI 2.60, 1.21–5.63); *p* value = 0.015), and urban residence ((CI 0.48, 0.30–0.78); *p* value = 0.003) were associated with successful induction of labor.

**Conclusion:**

These findings strongly suggest that cervical conditions are important determinants for the success of labor induction. Therefore, healthcare providers should confirm the favorability of the cervical status (using Bishop score) as a strict prerequisite before actual labor induction, and special consideration should be given to those pregnant women who reside in urban areas.

## 1. Introduction

Induction of labor (IOL) refers to the artificial initiation of uterine contractions once the fetus has reached viability and before the onset of spontaneous labor to enable vaginal delivery. It is one of the modern obstetric practices used to decrease the risk of maternal and neonatal morbidity and mortality by assisting the pregnancy to terminate, especially in the presence of a range of obstetric and medical conditions that threaten the continuation of pregnancy [1–3].

Induction of labor is one of the most frequently performed obstetric procedures in both developing and developed countries, aimed at initiating uterine contractions by surgical, mechanical, and/or medical means to enhance the likelihood of a normal vaginal delivery. Unlike the medical means of induction, which include oxytocin and prostaglandin analogs, the surgical and mechanical means of induction include artificial rupture of membranes, balloon catheters, and/or laminaria, respectively [4].

Induction of labor can be classified as either elective or emergency depending on maternal, fetal, social, or a combination of these factors. Elective induction is usually conducted with prior planning by the healthcare provider and the mother when continuing the pregnancy beyond a certain number of weeks, causing a risk to the mother or the fetus. Pregnancy-induced hypertension, prelabor rupture of the membrane (PROM), gestational diabetes mellitus (GDM), prolonged pregnancy (pregnancy beyond 42 weeks of gestation), and/or mild intrauterine growth retardation (IUGR) are some of the indications for elective procedures of induction. On the other hand, emergency induction is undertaken when there is an emergency maternal and fetal condition that necessitates immediate delivery, which is included immediately after the occurrence of prolonged PROM, severe IUGR, chorioamnionitis, intrauterine fetal death (IUFD), severe abruption placentae, congenital anomalies, postterm pregnancy, and severe preeclampsia and eclampsia [5, 6].

The decision to induce labor is seriously considered and taken when the risk of continuing with the pregnancy outweighs the benefits to the woman and/or the fetus [7, 8]. According to the World Health Organization's (WHO) recommendations, the practice of IOL should be performed only when there is a clear medical indication for it, and the expected benefits outweigh the potential harm [9].

Although induction of labor is a critical life-saving intervention that potentially reduces adverse pregnancy outcomes [10], sometimes it has adverse consequences for the health of the mother and/or the fetus. Postpartum hemorrhage, intrauterine infection, abnormal fetal heart rate patterns, increased costs, uterine rupture, maternal water intoxication, delivery of a preterm infant due to incorrect estimation of dates, high cesarean delivery and operative vaginal delivery rates with the risk of traumatic birth, and possible cord prolapse are some of the adverse consequences of IOL [1, 10, 11].

Whatever the outcome of IOL, the rates of IOL vary from region to region. According to the WHO reports, there are increasing numbers of pregnant women experiencing IOL in developing countries, with overall rates exceeding 20% of all births [11]. In developed countries, up to 25% of all deliveries at term now involve IOL [9].

In Ethiopia, IOL is practiced widely in all hospitals; however, the pooled prevalence of failed labor induction is high [12]. Although some pieces of evidence are available in Ethiopia regarding induction outcomes, all were conducted using secondary data from card reviews [1, 6, 13–15]. Assessing the determinants of successful IOL using primary data would help to improve the failure rate of IOL and its complications.

Hence, this study aimed to assess the outcomes and associated factors of labor induction. This research sought to address the gap in the lack of sufficient evidence regarding outcomes and associated factors for labor induction by using primary data among mothers who were managed at East Gojjam zone public hospitals in northwest Ethiopia.

## 2. Methods and Materials

### 2.1. Study Area and Period

The study was conducted to determine the outcome of labor induction in women who underwent IOL at East Gojjam zone public hospitals in northwest Ethiopia from February 25 to May 25, 2020. East Gojjam zone is one of the third most populous zones in Amhara region, Ethiopia. Debre Markos town is the capital city of East Gojjam zone, 300 km away from Addis Ababa in northwest Ethiopia. The zone has ten hospitals (one referral hospital, one general hospital, and eight primary hospitals), 104 health centers, and 406 health posts. According to the 2019 East Gojjam zone admiration office report, this zone had an estimated 91,634 women of reproductive age [16, 17].

### 2.2. Study Design

An institution-based prospective cross-sectional study design was employed.

### 2.3. Source and Study Population

All pregnant women who were admitted for induction of labor in East Gojjam zone public hospitals were taken as a source population, while those pregnant women who were admitted for induction of labor during the study period were considered as a study population. Pregnant mothers who underwent the induction of labor but were unable to communicate during a face-to-face interview because of convulsions or coma secondary to eclampsia were excluded.

### 2.4. Sample Size Determination

The sample size was calculated using a single population proportion formula by considering the following assumptions:

(1) $$\begin{array}{c} n=\frac{\left[{\left(Z\;a/2\right)}^{2}*p\left(1-p\right)\right]}{d^{2}}\text{,} \end{array}$$

where *n* = the minimum required sample size, *p* = prevalence of the success rate of induction (57.89%) taken from the previous study conducted in Oromia, Ethiopia [6], *d* = maximum tolerable error, which is = 5%, and *Z* (*a*/2) = value of the standard normal distribution at the 95% confidence level, which is 1.96:

(2) $$\begin{array}{c} n=\frac{\left(1.96\right)2\;x\left(0.58\right)\left(1-0.58\right)}{\left(0.05\right)2}=374\text{.} \end{array}$$

After adding a 10% nonresponse rate, the final sample size was 411.

### 2.5. Sampling Technique and Procedure

There are ten public hospitals in East Gojjam zone, and all public hospitals were included in the study. The average expected total number of pregnant mothers admitted for induction across the ten hospitals was 878, and this was used to determine the sampling interval. Then, the sample size was proportionally allocated for each hospital. Finally, by using the systematic random sampling technique, 411 participants were selected for every two pregnant women within a three-month period.

### 2.6. Data Collection Tool and Procedure

Data were collected using an interviewer-administered, structured, and pretested questionnaire, both from mothers and their medical charts. The questionnaire was developed by reviewing different previous studies. The questionnaire includes sociodemographic characteristics, obstetric history, details of IOL, and its outcomes (indications, methods, and mode of delivery). First, the questionnaire was prepared in English and translated into Amharic (the local language) and back to English by language experts. Before actual data collection, a pretest was performed by taking 5% of the study population at Finote Selam Primary Hospital, and necessary modifications were made accordingly.

### 2.7. Operational Definitions

Outcomes of labor induction refer to the result of labor induction, whether successful or unsuccessful, irrespective of indications and methods. The hospitals in the study area used a low-dose oxytocin infusion regimen.

#### 2.7.1. Inadequate Uterine Contractions

Uterine contractions between 3 and 5 within 10 minutes, each lasting 40–60 seconds, are not achieved despite being on an oxytocin drip for at least six to eight hours [18, 19].

#### 2.7.2. Failed Induction of Labor

Failed induction of labor is defined as failure to achieve a vaginal delivery following labor induction [13, 20, 21].

The Bishop score was measured and categorized as favorable (score ≥ 9), intermediate (score 5–8), or unfavorable (score < 4) [4].

### 2.8. Data Management and Analysis

The data were collected by trained BSc midwives not currently employed at the institutions in which they were performing data collection. A one-day training and a clear orientation were given for data collectors and supervisors. Also, the completeness of the data was checked by the data collectors, supervisors, and principal investigators on a daily basis. The data were manually cleaned and entered into Epi-Data TM Version 3.1. Further analysis was conducted using Stata/se™ Version 14 statistical software. Descriptive statistics were used to present and summarize the data in the form of frequency, percentage, and mean and standard deviation in tables with 95% confidence intervals for prevalence estimates.

Binary logistic regression was carried out to identify possible determining factors for the outcomes of IOL. Variables with a *p* value <0.25 in the bivariable logistic regression model were entered into a multivariable logistic regression model. In the multivariable analysis, variables with *p* values less than 0.05 and adjusted odds ratios with their 95% CIs were used to declare their statistical significance. The model's fitness was checked using the Hosmer and Lemeshow goodness of fit test, and multicollinearity among independent variables was checked using the variance inflation factors.

## 3. Results

### 3.1. Sociodemographic Characteristics

A total of 411 mothers were enrolled in this study, giving it a response rate of 100%. The mean age of the respondents was 28.56 (±5.60) years. Over half (57.1%) of the respondents were found within the age group of 25–34 years; of them, 172 (41.8%) experienced successful IOL. Of the total 411 respondents, 99.5% and 91.7% were from the Amhara ethnic group and Orthodox religion followers, respectively.

This finding indicated that nearly one-fourth of the participants were unable to read and write; of those, 83 (20.2%) had successful labor induction. Moreover, in this study, approximately one-fifth of the mothers were housewives, of which 59 (14.4%) delivered with successful labor induction (Table 1).

### 3.2. Obstetric Characteristics and Indications for IOL

Of the total study participants, 217 (52.8%) were primigravida, and among those, 1,143 (34.8%) had successful IOL. More than half (57.4%) of the mothers had at least one antenatal care (ANC) follow-up during the current pregnancy, of which successful labor induction was observed among 153 (37.2%) of the participants. In this study, the proportion of preterm IOL was 22.6%, and of those, IOL was successful in nearly one-fifth (19.5%) of the cases.

Regarding the indication for IOL, 31.4% of the mothers were induced due to postterm pregnancy, and from this, 81 (19.7%) of the inductions were successful. Premature rupture of membranes and pregnancy-induced hypertension were the second and third indications for IOL, respectively. Furthermore, more than one-fourth (26.3%) of the participant mothers were induced by oxytocin alone; among them, 58 (14.2%) of the inductions were successful (Table 2).

### 3.3. Outcomes of Labor Induction

This study revealed that, of all mothers who underwent IOL during the study period, 70.3% (65.6, 74.7) delivered vaginally (i.e., a successful outcome), followed by 122 (29.7%) by cesarean section (CS) for failed IOL. Over three-fourths (76.6%) of the respondents achieved adequate uterine contractions within six to eight hours of induction. The common indication for cesarean section (C/S) was failed induction in 78.7% of the cases, followed by fetal distress in 10.7% of the cases (Table 3).

### 3.4. Factors Associated with Successful Induction of Labor

In the bivariable logistic regression model, the favorable Bishop score, the intermediate Bishop score, gravidity, membrane rupture before induction, induction using oxytocin alone, and place of residence were eligible for multivariable analysis with a *p* value of less than 0.25. In the final multivariable logistic regression model, mothers who had favorable Bishop scores were 3.9 times more likely to experience successful induction ((CI 3.90, 1.63–9.29); *p* value = 0.002), mothers induced using oxytocin with cervical ripening were 2.6 times more likely to result in successful IOL than those induced by oxytocin only ((CI 2.60, 1.21–5.63); *p* value = 0.015), and urban women were about 52% less likely to experience successful labor induction than their rural counterparts ((CI 0.48, 0.30–0.78); *p* value = 0.003) (Table 4).

## 4. Discussion

Although induction of labor is a critical life-saving intervention that reduces adverse maternal and neonatal outcomes, sometimes it has adverse consequences for the health and wellbeing of the woman and/or the fetus [10]. Understanding the determinants of successful IOL using primary data would help to improve the failure rate of IOL and its complications.

In this study, the basic indications for IOL were postterm pregnancy, PROM, pregnancy-induced hypertension, and IUFD, which were 31.4%, 24.6%, 24.6%, and 12.7%, respectively. Successful IOL was achieved in 70.3% of the study participants. This finding is in line with previous studies conducted in Adama Hospital, Ethiopia (70.4) [22], Congo (70.1%) [23], and Ogoja (75.9%), Nigeria [24].

In the current study, the proportion of successful IOL was higher than in the findings reported from Lemlem Karl Hospital in Miachew town (54.5%), Wolliso St. Luke, Catholic Hospital (57.89%), and Wolaita Sodo (59.7%) [2, 6, 25]. This discrepancy may be due to the variations in procedures and indications for IOL. In a study conducted at Army Referral Hospital, direct oxytocin intravenous infusion was the only method used for the purpose of induction and premature rupture of membranes was the predominant indication for IOL. In this study, postterm pregnancies were the most common reason for IOL, which may provide a chance for cervical repining before actual induction and could increase its success. Also, the sociodemographic and the time variations between the current and previous studies may be a possible reason for this discrepancy.

However, the prevalence of successful induction in the current study was lower in comparison with the previous studies conducted at the University of Gondar Specialized Hospital (75.6%) [13], Jimma University Specialized Hospital (78.6%) [1], public hospitals of Mekelle town (76%) [14], Worabe Comprehensive Specialized Hospital (77.8%) [15], Nepal (76.5%) [11], Nigeria (75.9%) [24, 26], and Saudi Arabia (84%) [27]. This difference could be due to inductions for indication, with PROM being the most common reason for IOL in Hawassa and Gondar, whereas in Nigeria and Saudi Arabia, IOL began with cervical ripening and oxytocin IV infusion, which may increase the success of induction.

The current study shows that the odds of successful IOL decreased by 52% in mothers who resided in urban areas when compared to mothers from rural areas. According to various pieces of evidence, urbanization was significantly associated with chronic comorbidities such as increased BMI and others, which in turn were associated with macrosomia and an unfavorable pelvis, resulting in low induction success rates. Furthermore, the sociodemographic results from this study revealed that the majority of the participants, who lived in rural areas, were multiparous; this might explain why rural women had a higher success rate with induction. Different pieces of evidence revealed that increasing maternal parity is a strong indicator of the likelihood of successful IOL.

In addition, pregnant women with favorable Bishop scores before induction increase the success rate of induction. This finding is supported by studies conducted in Ethiopia and Pakistan [1, 28, 29]. With a favorable Bishop score, the success of IOL is similar to a spontaneous vaginal delivery as it reflects the normal changes the cervix undergoes in parturition. If the cervix is favorable, IOL is likely to result in a vaginal delivery.

Furthermore, pregnant women who were induced using oxytocin with cervical ripening methods nearly tripled the success of induction compared to those undergoing induction using oxytocin alone. In pregnant women whose cervix is unfavorable, cervical ripening using prostaglandin analogs markedly enhances the success of induction rather than the direct use of oxytocin alone [30, 31]. Generally, it is known that cervical conditions are important determinants for the success of labor induction. Therefore, cervical ripening is a prerequisite for the success of induction, specifically in the instance of an unfavorable Bishop score.

## 5. Conclusion

In this study, the overall success of IOL was found in about 70.3% of cases, which is considerably lower than the success rates in other Ethiopian studies. The favorable Bishop score, the intermediate Bishop score, induction using oxytocin with cervical ripening, and urban residence were significant factors associated with successful labor induction. Therefore, to improve the induction failure rate and its complications, healthcare providers should confirm the favorability of the cervical status as a strict prerequisite before actual labor induction, and special consideration should be given to those pregnant women who reside in urban areas.

## Figures and Tables

**Table 1:** Sociodemographic characteristics of mothers who delivered by induction of labor at East Gojjam public hospitals, northwest Ethiopia, 2020 (*n* = 411).

Variables	Categories	Outcomes of labor induction		Total *n* (%)
		Successful *n* (%)	Failed *n* (%)	
Age of the mother	15–24	73 (17.8)	34 (8.2)	107 (26.0)
	25–34	172 (41.8)	63 (15.3)	235 (57.2)
	>34	44 (10.7)	25 (6.1)	69 (16.8)

BMI	<25	180 (43.8)	56 (13.6)	236 (57.4)
	25–29.9	105 (25.5)	63 (15.4)	168 (40.9)
	≥30	4 (1.0)	3 (0.7)	7 (1.7)

Marital status	Married	285 (69.3)	122 (29.7)	407 (99.0)
	Divorced/widowed	4 (1.0)	0 (0.0)	4 (1.0)

Residence	Urban	143 (34.8)	81 (19.7)	224 (54.5)
	Rural	146 (35.5)	41 (10.0)	187 (45.5)

Educational status	Unable to read	83 (20.2)	20 (4.9)	103 (25.1)
	Able to read and write	25 (6.1)	11 (2.6)	36 (8.7)
	Primary school	71 (17.3)	25 (6.1)	96 (23.4)
	Secondary school	57 (13.9)	35 (8.5)	92 (22.4)
	Diploma and above	53 (12.9)	31 (7.5)	84 (20.4)

Maternal occupation	Farmer	111 (27.0)	29 (7.1)	140 (34.1)
	Merchant	70 (17.0)	46 (11.2)	116 (28.2)
	Housewife	59 (14.4)	24 (5.8)	83 (20.2)
	Government employee	48 (11.6)	22 (5.4)	70 (17.0)
	No work	1 (0.2)	1 (0.25)	2 (0.5)

**Table 2:** Obstetric and induction characteristics of women who delivered by induction of labor at East Gojjam zone public hospitals, northwest Ethiopia, 2020 (*n* = 411).

Variables	Categories	Successful labor induction		Total *n* (%)
		Yes *n* (%)	No *n* (%)	
Gravidity	Primigravida	143 (34.8)	74 (18.0)	217 (52.8)
	Multigravida	146 (35.5)	48 (11.7)	194 (47.2)

Parity	Primiparous	157 (38.2)	76 (18.5)	233 (56.7)
	Multipara	132 (32.1)	46 (11.2)	178 (43.3)

Gestational age at induction	<36^+6^ weeks	80 (19.5)	13 (3.1)	93 (22.6)
	37–41^+6^ weeks	115 (28.0)	57 (13.8)	172 (41.8)
	≥42 weeks	90 (21.9)	50 (12.2)	140 (34.1)
	Unknown LMP	4 (1.0)	2 (0.5)	6 (1.5)

ANC	Yes	153 (37.2)	83 (20.2)	236 (57.4)
	No	136 (33.1)	39 (9.5)	175 (42.6)

Indications for induction	Postterm pregnancy	81 (19.7)	48 (11.7)	129 (31.4)
	Prelabor rupture of membranes	58 (14.1)	43 (10.5)	101 (24.6)
	Intrauterine fetal death	48 (11.7)	4 (1.0)	52 (12.7)
	Pregnancy-induced hypertension	79 (19.2)	22 (5.4)	101 (24.6)
	Others^*∗*^	23 (5.6)	5 (1.2)	20 (6.8)

Membrane rupture before induction	Yes	70 (17.0)	46 (11.2)	116 (28.2)
	No	219 (53.5)	76 (18.3)	295 (71.8)

Method of induction	Oxytocin alone	58 (14.2)	50 (12.1)	108 (26.3)
	Oxytocin with cervical ripening	231 (56.2)	72 (17.5)	303 (73.7)

Bishop score	≥9	33 (8.0)	8 (2.0)	41 (10.0)
	5–8	183 (44.5)	43 (10.5)	226 (55.0)
	<4	73 (17.8)	71 (17.2)	144 (35.0)

NB: Others ^*∗*^: antenatal hemorrhage, spinal bifida, amniotic fluid disorders, hydrocephaly, and anencephaly; LMP: last menstrual period.

**Table 3:** Outcomes of labor induction in East Gojjam zone public hospitals, northwest Ethiopia, 2020.

Variable	Categories	Frequency	Percentage (%)
Mode of delivery	Vaginal	289	70.3
	C/S	122	29.7

Adequate uterine contraction	Yes	315	76.6
	NO	96	23.4

C/S indications	Inadequate uterine contraction	96	78.7
	NRFHP	13	10.7
	CPD	11	9.0
	Others^*∗*^	2	1.6

Complications of induction	Uterine hyperstimulation	4	21.05
	NRFHP	15	78.95

NB: Others ^*∗*^: cervical arrest and face presentation; CPD: cephalopelvic disproportion; C/S: cesarean section; NRFHP: nonreassuring fetal heart rate pattern.

**Table 4:** Factors associated with successful induction of labor among mothers who delivered at East Gojjam zone public hospitals, northwest Ethiopia, 2020 (*n* = 411).

Variables	Categories	Successful IOL		Odds ratio (95% CI)		*p* value
		Yes	No	COR	AOR	
Residence	Urban	143	81	0.50 (0.32–0.77)	0.48 (0.30–0.78)	0.003
	Rural	146	41	1	1	

Gravidity	Primigravida	143	74	0.64 (0.41–0.98)	0.76 (0.47–1.21)	0.246
	Multigravida	146	48	1	1	

PROM before IOL	Yes	70	46	0.53 (0.34–0.83)	1.38 (0.63–3.01)	0.423
	No	219	76	1	1	

Bishop score	Favorable	33	8	4.01 (1.73–9.28)	3.90 (1.63–9.29)	0.002
	Intermediate	183	43	4.14 (2.60–6.60)	3.53 (2.15–5.82)	0.001
	Unfavorable	73	71	1	1	

Induction methods	Oxytocin alone	58	50	1	1	
	Oxytocin with cervical ripening	231	72	2.77 (1.74–4.39)	2.60 (1.21–5.63)	0.015

NB: IOL: induction of labor; PROM: premature rupture of membranes; 1 = reference; Hosmer and Lemeshow test for the adjusted model: chi-square = 7.68, *p*=0.36.

## Data Availability

The datasets used and/or analyzed during the study are available from the corresponding author (GTM) on reasonable request.

## Abbreviations

ANC: Antenatal care
AOR: Adjusted odds ratio
BMI: Body mass index
C/S (C/D): Cesarean section/delivery
IOL: Induction of labor
IUFD: Intrauterine fetal death
IUGR: Intrauterine growth restriction
PROM: Premature rupture of membranes.

## References

[1] Girma W., Tseadu F., Wolde M. (2016). Outcome of induction and associated factors among term and post-term mothers managed at Jimma University specialized hospital: a two years’ retrospective analysis. *Ethiopian journal of health sciences*.

[2] Rade B., Mitku Y., Weldemicheal A., Zenebe Z., Desalegn A., Bitsu B. (2018). Induction of labor and its determinant factors: retrospective cross-sectional study from a public hospital in Ethiopia. *Journal of Pregnacy and Child Health*.

[3] Tandu-Umba B., Tshibangu R. L., Muela A. M. (2013). Maternal and perinatal outcomes of induction of labor at term in the university clinics of Kinshasa, DR Congo. *Open Journal of Obstetrics and Gynecology*.

[4] Beshir Y. M., Kure M. A., Egata G., Roba K. T. (2021). Outcome of induction and associated factors among induced labours in public Hospitals of Harari Regional State, Eastern Ethiopia: a two years’ retrospective analysis. *PLoS One*.

[5] Laughon S. K., Zhang J., Grewal J., Sundaram R., Beaver J., Reddy U. M. (2012). Induction of labor in a contemporary obstetric cohort. *American Journal of Obstetrics and Gynecology*.

[6] Abdulkadir Y., Dejene A., Geremew M., Dechasa B. (2017). Induction of labor prevalence and associated factors for its outcome at Wolliso St. Luke. Catholic hospital, south west shewa, Oromia. *Internal Medicine Inside*.

[7] Alkasseh A. (2019). Oral misoprostol as an alternative labor induction method: a case control study. *Clinics in Mother and Child Health*.

[8] Mbele A., Makin J. D., Pattinson R. C. (2007). Can the outcome of induction of labour with oral misoprostol be predicted?. *South African Medical Journal*.

[9] Organization W. H. (2011). *WHO Recommendations for Induction of Labour*.

[10] Tenagnework Dilnessa KtaAW (2019). The proportion of failed induction of labour and associated factors among women undergoing induction of labour in dessie referral hospital: northeast Ethiopia a cross-sectional study. *Asian Journal of Pregnancy and Childbirth*.

[11] Acharya T., Devkota R., Bhattarai B., Acharya R. (2017). Outcome of misoprostol and oxytocin in induction of labour. *SAGE open medicine*.

[12] Melkie A., Addisu D., Mekie M., Dagnew E. (2021). Failed induction of labor and its associated factors in Ethiopia: a systematic review and meta-analysis. *Heliyon*.

[13] Tadesse T., Assefa N., Roba H. S., Baye Y. (2022). Failed induction of labor and associated factors among women undergoing induction at University of Gondar Specialized Hospital, Northwest Ethiopia. *BMC Pregnancy and Childbirth*.

[14] Lueth G. D., Kebede A., Medhanyie A. A. (2020). Prevalence, outcomes and associated factors of labor induction among women delivered at public hospitals of MEKELLE town-(a hospital based cross sectional study). *BMC Pregnancy and Childbirth*.

[15] Mohammed M., Oumer R., Mohammed F. (2022). Prevalence and factors associated with failed induction of labor in Worabe comprehensive specialized hospital, southern Ethiopia. *PLoS One*.

[16] Dmzh O. (2019). *Annual Plan and Programme of East Gojjam Zonal Health Office Report Zonal Health Office*.

[17] Agmassie G. A., Alamneh G. D., Ayicheh M. W., Getahun W. T., Abneh A. A. (2023). The magnitude and associated factors of immediate postpartum anemia among women who gave birth in east Gojjam zone hospitals, northwest- Ethiopia, 2020. *PLoS One*.

[18] Tesemma M. G., Sori D. A., Hiko D. (2020). High dose and low dose oxytocin regimen as determinants of successful induction: a multicenter comparative study. *BMC Pregnancy Childbirth*.

[19] Gebreyohannes R. D., Mesfin E. (2020). Determinants of outcome of induction of labor in four teaching hospitals in Addis Ababa, Ethiopia. *Ethiopian Medical Journal*.

[20] Debele T. Z., Cherkos E. A., Badi M. B. (2021). Factors and outcomes associated with the induction of labor in referral hospitals of Amhara regional state, Ethiopia: a multicenter study. *BMC Pregnancy and Childbirth*.

[21] Frederiks F., Lee S., Dekker G. (2012). Risk factors for failed induction in nulliparous women. *Journal of Maternal-Fetal and Neonatal Medicine*.

[22] Demssie E. A., Deybasso H. A., Tulu T. M., Abebe D., Kure M. A., Teji Roba K. (2022). Failed induction of labor and associated factors in Adama hospital medical College, Oromia regional state, Ethiopia. *SAGE Open Medicine*.

[23] Tandu-Umba B., Tshibangu R. L., Muela A. M. (2013). *Maternal and Perinatal Outcomes of Induction of Labor at Term in the university Clinics of Kinshasa*.

[24] Lawani O. L., Onyebuchi A. K., Iyoke C. A., Okafo C. N., Ajah L. O. (2014). Obstetric outcome and significance of labour induction in a health resource poor setting. *Obstetrics and gynecology international*.

[25] Bekru E. T., Yirdaw B. E. (2018). Success of Labour Induction Institution Based Cross-Sectional Study Wolaita Sodo, South Ethiopia. *International Journal of Nursing and Midwifery*.

[26] Oyebode T. A., Toma B. O., Shambe I. H. (2015). Induction of labour at jos university teaching, hospital, jos, nigeria: a four year review. *International Journal of Research in Medical Sciences*.

[27] Al-Shaikh G. K., Wahabi H. A., Fayed A. A., Esmaeil S. A., Al-Malki G. A. (2012). Factors associated with successful induction of labor. *Saudi Medical Journal*.

[28] Khan N. B., Ahmed I., Malik A., Sheikh L. (2012). Factors associated with failed induction of labour in a secondary care hospital. *JPMA. The Journal of the Pakistan Medical Association*.

[29] Hurissa B., Geta M., Belachew T. (2015). Prevalence of failed induction of labor and associated factors among women delivered in Hawassa public health facilities, Ethiopia, 20. *Journal of Women’s Health Care*.

[30] Harman J. H., Kim A. (1999). Current trends in cervical ripening and labor induction. *American Family Physician*.

[31] Tenore J. L. (2003). Methods for cervical ripening and induction of labor. *American Family Physician*.

